# Arginine Vasopressin and Copeptin in Perinatology

**DOI:** 10.3389/fped.2016.00075

**Published:** 2016-08-02

**Authors:** Katrina Suzanne Evers, Sven Wellmann

**Affiliations:** ^1^Division of Neonatology, University of Basel Children’s Hospital (UKBB), Basel, Switzerland

**Keywords:** antidiuretic hormone, HPA axis, asphyxia, respiratory distress, pain, stress, cesarean section, neonate

## Abstract

Arginine vasopressin (AVP) plays a major role in the homeostasis of fluid balance, vascular tonus, and the regulation of the endocrine stress response. The measurement of AVP levels is difficult due to its short half-life and laborious method of detection. Copeptin is a more stable peptide derived from the same precursor molecule, is released in an equimolar ratio to AVP, and has a very similar response to osmotic, hemodynamic, and stress-related stimuli. In fact, copeptin has been propagated as surrogate marker to indirectly determine circulating AVP concentrations in various conditions. Here, we present an overview of the current knowledge on AVP and copeptin in perinatology with a particular focus on the baby’s transition from placenta to lung breathing. We performed a systematic review of the literature on fetal stress hormone levels, including norepinephrine, cortisol, AVP, and copeptin, in regard to birth stress. Finally, diagnostic and therapeutic options for copeptin measurement and AVP functions are discussed.

## Introduction

The nature of stress hormones is comparable to that of firefighters: they act fast on demand and pull back as soon as possible. While firefighters return to the firehouse for recreation once their task is finished, the activity of stress hormones is naturally limited due to their short half-life, which allows for a targeted and time-limited action but complicates their detection in the circulatory system. One such stress hormone is arginine vasopressin (AVP), also known as antidiuretic hormone (ADH), which is a small peptide with a multitude of functions in many systems, including the central nervous system (CNS). Fortunately, AVP is derived together with three other peptides from a larger precursor peptide, and one of these peptides, copeptin, is more stable than AVP itself and is released in a 1:1 ratio to AVP. One decade ago, a quantitative copeptin sandwich immunoassay was developed (1), and since then, a large variety of clinical studies demonstrated the usefulness of copeptin to indirectly determine circulating AVP concentrations.

This review briefly summarizes the basic information on AVP and copeptin in general, including their production and action *via* AVP receptors, followed by analysis of the relationship between labor and stress hormone release and the action of AVP in healthy neonates and in newborn diseases. The particular focus of this review is on the role of AVP in the transition from placenta to lung breathing and in acute and chronic stress responses. We performed a systematic review of the literature on various stress hormones at birth, such as norepinephrine, cortisol, AVP, and copeptin, and present them here. Finally, the therapeutic and diagnostic avenues of AVP and copeptin are outlined.

## Production of AVP and Copeptin

Arginine vasopressin is produced as a larger precursor pre-proAVP by magnocellular and parvocellular neurons within the paraventricular nucleus (PVN) and the supraoptic nucleus (SON) of the hypothalamus (2). Pre-proAVP is produced by magnocellular neurons of the PVN and SON, is packaged into neurosecretory granules, and is transported axonally to the posterior pituitary, also called the neurohypophysis. En route, pre-proAVP is enzymatically processed into four peptides: the N-terminal signal peptide, the active hormone AVP, neurophysin 2, and the C-terminal copeptin. Upon activation with various stimuli, the stored peptides AVP, neurophysin 2, and copeptin are secreted into the circulatory system in equimolar amounts. The pre-proAVP produced in parvocellular neurons in the PVN is transported by axons projecting to the median eminence where it is processed and secreted into the hypothalamic–hypophysial portal vessels and ultimately reaches its destination, the anterior pituitary. A third portion is produced by parvocellular neurons of the PVN, the medial amygdala, the bed nucleus of the stria terminalis, and the suprachiasmatic nucleus, with projections toward distinct brain regions (3). Together, there are at least three distinct pathways by which AVP exerts its functions. First, AVP regulates water absorption *via* the posterior pituitary. Second, AVP is critically involved in the hypothalamic-pituitary-adrenal (HPA) stress axis *via* the posterior pituitary. Third, AVP remaining in the CNS contributes to behavior and cognitive functions.

## AVP Receptors

The receptors for AVP have been divided into three major types, V1a, V1b (or V3), and V2, according to their pharmacological and G-protein-coupled properties (2). AVP released into the circulatory system functions as a peripheral hormone by binding to its receptors located at the plasma membrane of various target cells. The V1a receptor is predominantly found in vascular smooth muscle and is involved in the control of vasoconstrictor effects and blood pressure regulation. V1b receptors are primarily located on specialized cells, called corticotrophs, in the anterior pituitary gland, where they stimulate the release of adrenocorticotropic hormone (ACTH) synergistically with corticotropin-releasing hormone (CRH). The V2 receptor expressed on kidney cells is responsible for water reabsorption in the collecting ducts by activating aquaporin-2 channels, whereas its expression on endothelial cells of the vasculature and platelets makes AVP an important hormone in hemostasis. V1a and V1b receptors are found in the brain. Finally, AVP binds to the oxytocin receptor, which further increases its complexity (4).

## AVP and Copeptin Measurement

The measurement of AVP is cumbersome and complex due to several pre-analytical obstacles; thus, the detection of AVP is unsuited for clinical diagnostics and is limited to a few specialized laboratories (5). For example, 90% of AVP in the circulatory system is bound to platelets, which falsifies the actual amounts of circulating AVP (6). AVP is a bioactive peptide hormone that is tightly regulated and rapidly cleared from the circulation, with an *in vivo* half-life of less than 30 min (7). And to make matters worse, AVP is unstable in isolated plasma, even when stored at −20°C (8). In contrast, copeptin does not have such limitations (5). Several copeptin assays are currently available, but the only assays with sufficient technical descriptions and clinical data to justify their routine clinical use are the original sandwich immunoluminometric assay (1) and its automated immunofluorescent successor (on the KRYPTOR platform). In conclusion, the great advantages of copeptin measurement are the remarkably high sensitivity of this robust AVP surrogate marker, its extreme stability once collected in blood sampling tubes, and the fact that only 50 μl of serum or plasma are needed for the assay (9).

## Surge in Fetal Stress Hormones

An important “side effect” of vaginal delivery is the surge in fetal stress hormones, including catecholamines, cortisol, and AVP. These stress hormones are widely recognized to facilitate the transition of the newborn to air breathing, cardiovascular adaptation, thermogenesis, glucose, and water homeostasis. It has been four decades since the first results on AVP concentrations in an infant’s umbilical cord blood at birth were published, which indicated strongly elevated AVP concentrations (10–16). These initial studies have led to the following key findings: (1) cord blood AVP is of fetal origin because AVP concentrations in arterial cord blood are higher than values reported in venous cord blood (arteriovenous gradient) and because AVP is absent in cord blood of anencephalic infants (10–12, 14, 16, 17), (2) cord blood AVP levels in infants delivered vaginally are much higher than those in infants delivered by cesarean section performed before the onset of labor (15, 16), (3) cord blood AVP levels correlate positively with fetal distress measured as cord blood pH (17) and have been reported to be exceedingly high in asphyxiated infants (10, 14, 18), leading to the assumption of “pituitary exhaustion” (10), and (4) following delivery, AVP levels drop rapidly within a few hours to adult basal levels (16, 19).

With the goal to study systematically the surge of AVP and copeptin levels at birth in comparison to other stress hormones, namely cortisol and norepinephrine, we performed a systematic review of the literature. The authors independently searched the database Medline, available *via* PubMed, retrieved, and reviewed studies for eligibility and extracted data. All disagreements encountered in the review process were resolved through consensus. We included studies that met the following criteria: (1) study design: cohort, cross-sectional, or case-control studies in humans, which evaluated the relationship between different delivery modes and concentrations of stress hormones in healthy term-born infants at birth. Only studies comparing unassisted vaginal delivery with primary cesarean section were included. Studies were excluded from this review if they reported emergency or secondary cesarean sections, the latter are cesareans with contractions or rupture of the membranes, and if a study reported only data on either vaginal deliveries or primary cesarean sections. (2) Outcome measures: plasma or serum concentrations of cortisol, norepinephrine, AVP, and copeptin in arterial or venous umbilical cord blood collected at birth. (3) Statistical analysis: the authors of the studies must have presented hormone concentrations as medians or means as well as patient numbers of each delivery group separately. Searches were carried out to identify studies for each hormone concerned with: (1) neonate, (2) umbilical cord blood, and (3) delivery or birth. These three concepts were linked by the “AND” operator. A variety of search terms was used within each concept, e.g., (1) newborn, baby, infant, fetus; (2) cord blood, serum, plasma; and (3) parturition, delivery mode, birth stress. Both free text and subject headings (MeSH terms) were used.

**Figure 1:**
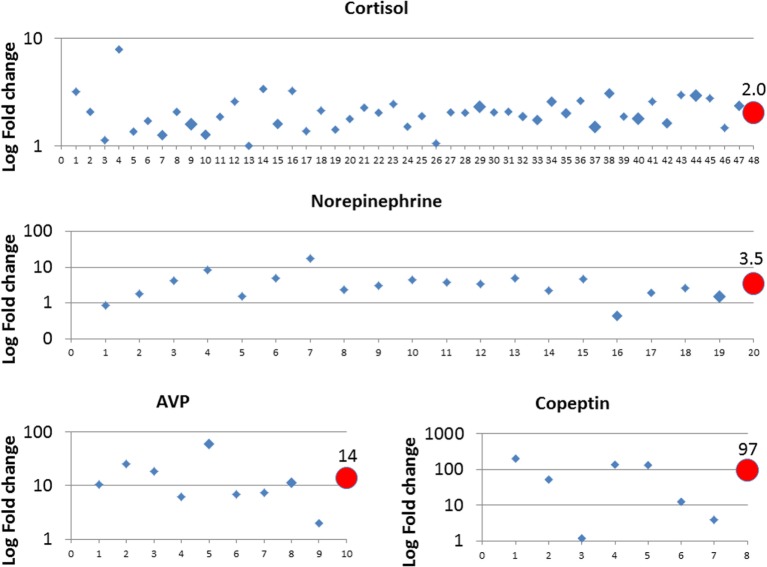
**Fold change of norepinephrine, cortisol, AVP, and copeptin between primary cesarean section and vaginal delivery**. Study data were retrieved from the published literature and listed in chronologic order from oldest to newest on *x*-axis, corresponding references are given in Section “Surge in Fetal Stress Hormones” for all four hormones. The size of the diamonds reflects the study sample size: small corresponds to *n* = 0–50, middle *n* = 50–100, and large *n* = 100–170. Circles reflect the weighted mean values depending on sample size and are given at the final position of the *x*-axis.

For cortisol, 47 studies, norepinephrine 19, AVP 9, and for copeptin, 7 studies were eligible and data were retrieved. Data are presented in Figure 1 as fold change between vaginal delivery and primary cesarean section of cortisol, norepinephrine, AVP, and copeptin in separate panels. For each hormone, a mean fold change is given weighted with respect to sample size. Studies are numbered chronologically, and the corresponding references are as follows:

Cortisol: 1 (20), 2 (21), 3 (22), 4 (23), 5 (24), 6 (25), 7 (26), 8 (27), 9 (28), 10 (29), 11 (30), 12 (31), 13 (32), 14 (33), 15 (34), 16 (35), 17 (36), 18 (37), 19 (38), 20 (39), 21 (40), 22 (41), 23 (42), 24 (43), 25 (44), 26 (45), 27 (46), 28 (47), 29 (48), 30 (49), 31 (50), 32 (51), 33 (52), 34 (53), 35 (54), 36 (55), 37 (56), 38 (57), 39 (58), 40 (59), 41 (60), 42 (61), 43 (62), 44 (63), 45 (64), 46 (65), and 47 (66); Norepinephrine: 1 (67), 2 (68), 3 (33), 4 (69), 5 (70), 6 (71), 7 (72), 8 (73), 9 (74), 10 (36), 11 (75), 12 (76), 13 (77), 14 (78), 15 (79), 16 (80), 17 (81), 18 (55), and 19 (66); and AVP: 1 (11), 2 (12), 3 (15), 4 (16), 5 (82), 6 (83), 7 (41), 8 (84), and 9 (85). Copeptin: 1 (86), 2 (87), 3 (88), 4 (89), 5 (90), 6 (91), and 7 (92).

In summary, all four hormones were higher in the cord blood of infants after vaginal delivery than after primary cesarean section, with highest values observed for AVP (14-fold) and copeptin (97-fold). Of note, we identified a few studies that reported no difference (cortisol study number 13 and 26, copeptin study number 3) or lower values after vaginal delivery (norepinephrine study 1 and 16) (Figure 1). For the studies on cortisol and norepinephrine, we could not identify a specific reason. With respect to copeptin, five out of seven included studies conducted copeptin measurements using the validated BRAHMS KRYPTOR immunoanalyzer (Thermo Scientific Brahms GmbH, Hennigsdorf, Germany). Study number 3 used the human copeptin ELISA kit from Cusabio Inc. (Cusabio Biotech Co., USA) and study number 7 used the ELISA kit from Phoenix Pharmaceuticals Inc. (Phoenix Pharmaceuticals Inc., Karlsruhe, Germany), where both studies showed fold change below fivefold.

Both AVP and copeptin concentrations are significantly higher in infants after secondary cesarean section and assisted vaginal delivery as compared to primary cesarean and spontaneous delivery, respectively (11, 15, 16, 86, 90, 93). In addition, AVP and copeptin are closely related with metabolic stress markers pH, negative base excess, and lactate at birth, with highest concentrations after perinatal hypoxia ischemia, see also below (86, 87, 94, 95). Together, these findings support the pivotal role of AVP and copeptin as fetal stress markers.

## Labor and AVP and Copeptin Release

Birth is more than just the few minutes of delivery. It is a complex process that involves the preparation of the mother’s and infant’s bodies. Maternal labor is the driving force in vaginal delivery, as it encourages the mother’s body to dilate the cervix and to push the fetus through the birth canal (96). Normal labor is initiated by the removal of the inhibitory effects of pregnancy on the myometrium rather than an active process governed by uterine stimulants (97). The uterus is usually maintained in a state of quiescence during pregnancy. Myometrium inhibition occurs *via* the integrated action of different inhibitors, and their effect diminishes near term due to the activation of the uterus with regular uterine contractions accompanied by slow cervical dilation. This process represents the latent phase of the first stage of labor, which ultimately results in the descent of the fetal head into the pelvis. The second stage of labor begins when the cervix is fully dilated and ends with the delivery of the infant. Stage three events (uterine involution) occur after delivery. During the first and second stages of labor, each uterine contraction leads to a brief pO_2_ drop in the fetus, often without noticeable changes in fetal heart rate tracings (98–100). Data from animal studies show a direct and dose-dependent link between uterine contractions and fetal arterial pO_2_ changes (101, 102), as well as a relationship between arterial pO_2_ and plasma AVP in fetal sheep (103–105). Furthermore, AVP stimulates ACTH release and cortisol secretion, as demonstrated repeatedly in fetal sheep (106–109). Even small changes in fetal arterial pO_2_ of 2.5 mmHg were sufficient to cause ACTH release, a key hormone of the HPA axis downstream of AVP and CRH (110). Arterial hypoxia is a strong trigger of AVP (111) and copeptin release (112), and brief events of a few minutes of hypoxia are sufficient to cause a fivefold increase in copeptin levels (113). Very recently, we completed a randomized controlled trial with a truncated oxytocin challenge test 2 h prior to elective cesarean section with subsequent arterial umbilical cord blood sampling at birth. Plasma copeptin levels were found to be threefold higher in the newborn babies of the oxytocin group than in the placebo infants without any evidence of fetal acidosis (114). Interestingly, only half the women in the oxytocin group noticed the contractions, indicating that the mild subclinical contractions are sufficient to trigger fetal AVP/copeptin release.

Thus, we conclude that uterine contractions precondition the fetus by activating the fetal HPA axis hours and days before birth. AVP plays a key role in this cascade. After small fetal arterial pO_2_ changes due to uterine contractions, fetal AVP is released with subsequent ACTH and cortisol secretion.

## AVP in Healthy Neonates

**Figure 2:**
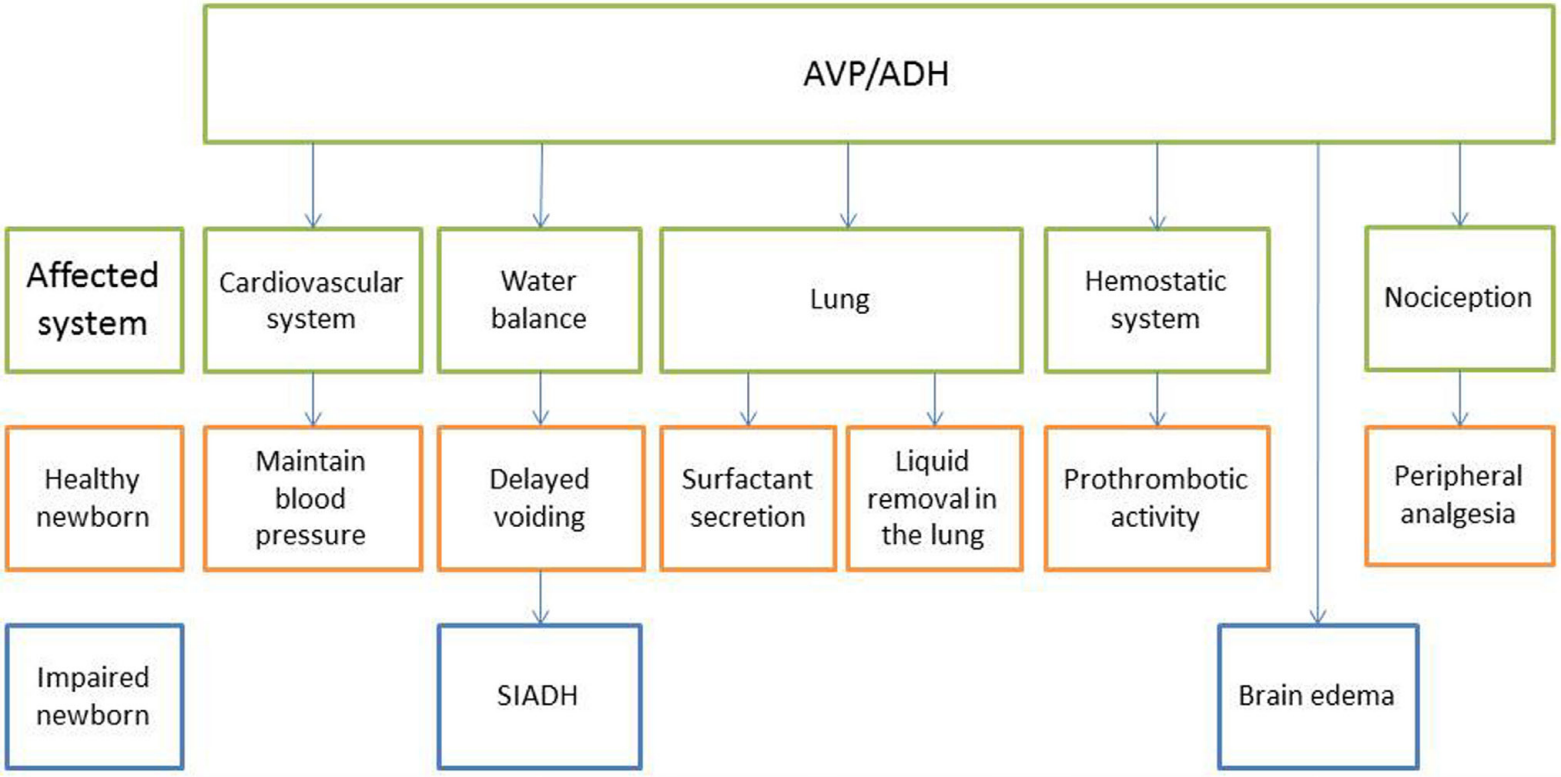
**Effects of AVP/ADH on various systems in the healthy and impaired newborn**.

Arginine vasopressin is related to a variety of transitional processes at birth, including water homeostasis, breathing, peripheral analgesia, regulation of the vasculature resistance, and platelet function, which are summarized below and shown in Figure 2.

### Body Fluid Homeostasis

Increased AVP levels at birth have been linked to delayed first voiding in newborns (93, 115), and copeptin concentrations at birth are inversely related to postnatal weight loss by day 3 of life (86). Reducing urinary water loss at birth until the enteral supply picks with the establishment of lactation appears to be a prudent strategy, and cesarean section without any previous labor deprives the newborn infant of the wave of fetal AVP, which serves as a preemptive adaptation. In fact, the extent of weight loss is much greater in newborns born by cesarean than by vaginal delivery (116, 117).

### Breathing

*In vitro* studies showed that cultivated type II pneumocytes express AVP receptor-2 and secrete phospholipoproteins after AVP binding, the constituents of surfactant (118, 119). Pulmonary surfactant is a surface-active phospholipoprotein complex, is produced by type II pneumocytes, and acts at the air–water interface of alveoli to reduce surface tension. Primary surfactant deficiency is a key feature in the pathogenesis of neonatal respiratory distress syndrome (RDS). Of interest, a pilot trial in extremely preterm infants to evaluate AVP versus dopamine as initial therapy in hypotension during the first 24 h of life showed that the AVP group received significantly fewer doses of surfactant and had lower PaCO_2_ values than the dopamine group (120). Compared to vaginal delivery, infants delivered by cesarean section have a significantly increased risk of neonatal admission because of respiratory complications mainly due to delayed lung fluid reabsorption after birth, namely wet lung (121). AVP has been shown to decrease the production of fetal lung liquid in ovine fetuses (122), and it has been shown that AVP is involved in liquid removal from the lungs at birth (123–125). Thus, it might be speculated that, in the absence of preconditioning, when primary cesarean section was performed, AVP administration shortly after birth to the baby, e.g., *via* nasal spray, might represent a novel strategy to support postnatal adaptation.

### Analgesia

Peripheral analgesia has been observed in vaginally delivered infants on the first day of life and has been linked to AVP signaling (4). In particular, animal studies have shown that the vasopressin-1A receptor mediates oxytocin and AVP-induced analgesia (126, 127).

### Cardiovascular System

Apart from water reabsorption in the kidney, vasoconstriction is a key function of AVP and has been reviewed elsewhere (128). Vasoconstriction is of vital importance to the neonate immediately after delivery to maintain blood pressure by increasing peripheral resistance and to close the umbilical cord blood vessels. AVP induces a redistribution of fetal blood flow from peripheral areas to vital organs (129). On a molar basis, AVP is even more potent than norepinephrine and angiotensin II (130). In contrast to the peripheral arterioles, AVP reduces pulmonary artery resistance through the stimulation of V1 receptors, which induces the release of the very potent vasodilator endothelial-derived nitric oxide (131). This opposing effect of AVP on the vascular bed is particularly advantageous in the transition from placenta to lung breathing and makes AVP a highly attractive drug in neonatology (120).

### Hemostasis

Arginine vasopressin stimulates the release of coagulation factor, von Willebrand factor (VWF), and tissue plasminogen activator (t-PA) *via* V2 receptors on endothelial cells and platelets. AVP constitutes an important factor in prothrombotic activity, and the AVP analog desmopressin (DDAVP) is widely used in a variety of bleeding diseases (132). Interestingly, thrombotic sealing is a crucial event for the closure of the ductus arteriosus, promoting vascular obliteration and subsequent remodeling (133). Apart from the absolute platelet count, platelet function may also play an important role in the pathobiology of the ductus arteriosus (134). Based on this finding, we speculate that AVP released at birth may contribute to the thrombotic sealing of the ductus arteriosus.

### Sex Difference

Both in healthy adults as well as in healthy neonates, AVP/copeptin plasma values are higher in males than in females (1, 90, 135–139).

## AVP in Newborn Diseases

When birth stress is increased because the fetus is compromised by prolonged hypoxic events, perinatal hypoxia ischemia (asphyxia) may result in which the highest fetal AVP and copeptin concentrations have been measured (87, 94, 95). While the difference between copeptin concentrations at birth of healthy neonates after elective cesarean delivery and vaginal delivery is about 100-fold (Figure 1), the difference between healthy vaginally delivered neonates and neonates with asphyxia is small (only twofold on average) (87). A major complication of asphyxia is brain edema, which may lead to increased intracranial pressure and is associated with poor outcome (140). The blood–brain barrier (BBB) Na/H exchanger is stimulated by brief exposure to hypoxia, aglycemia, and AVP (141) involving the V1 receptor, which is present in brain microvessels (142–144). Brain edema in animal models could be reduced by an AVP blocker (Conivaptan) (145, 146) and aggravated by AVP delivery (147, 148). Moreover, in the AVP-deficient Brattleboro rat, brain edema was attenuated compared to the control Long–Evans strain (149). Importantly, either intranasal AVP or AVP receptor antagonist did not evoke edema in the non-ischemic cortex of Mongolian gerbils (148). This result could indicate that increased AVP concentrations alone, namely after normal vaginal delivery, are insufficient to deteriorate the integrity of the BBB and that a severe lack of oxygen must be present as well.

A second sequela of asphyxia is the syndrome of inappropriate ADH secretion (SIADH), which is also a consequence of exceedingly high levels of AVP/ADH, renal failure, or both (150–154). Again, increased AVP concentrations alone, namely after normal vaginal delivery, have never been shown to cause SIADH, but they cause delayed voiding as described above.

Syndrome of inappropriate ADH secretion is also known as a complication of neonatal sepsis. In fact, sudden hyponatremia is a typical early sign in the early course of a severe neonatal infection. The pro-inflammatory cytokine interleukin-6 directly triggers the release of AVP (155, 156).

Chronic stress is associated with chronically elevated AVP/copeptin levels in rodent as well as human fetuses suffering from intrauterine growth restriction (82, 157, 158). After birth, neonates exposed to chronic stress, such as mechanical respiratory support, have elevated plasma copeptin concentrations (159).

## AVP in Therapy

Arginine vasopressin and its analogs have not yet been studied systematically in neonates (160). Various series of case reports indicate that AVP and its analogs are well tolerated and effective in neonates as rescue therapy for refractory arterial hypotension and particularly for persistent pulmonary hypertension (161–164). The recently published results of the first prospective randomized, double-blinded, pilot trial comparing AVP and dopamine in the treatment of hypotension in extremely low birth weight infants demonstrated the safety of AVP in these tiny infants (120). Although there were no differences between the two groups concerning time to adequate mean blood pressure, it was shown that infants who received vasopressin had lower mean PaCO_2_ levels and needed fewer doses of surfactant than infants who received dopamine; additionally, infants who received vasopressin did not show tachycardia during drug administration compared to infants who received dopamine. The authors believe that these effects may be due to the pulmonary vasodilatory effects and lack of cardiac effects of vasopressin (120).

## Conclusion

In view of the various actions AVP is involved (Figure 2) and the close relationship between fetal AVP and arterial pO_2_, we would like to conclude that the surge in fetal stress hormones prior to vaginal delivery is the beneficial result of intermittent hypoxic preconditioning of the fetus due to the uterine contractions required to dilate the cervix and to push the fetus through the birth canal.

In respect to diagnostic implications and the data presented here (Figure 1), copeptin is apparently the most sensitive birth stress biomarker when measured with a clinically established assay. Future research will investigate copeptin in the diagnosis of AVP-dependent disorders of fluid homeostasis in infants and children as well, including nephrogenic and cerebral reasons such as increased intracerebral pressure after intraventricular hemorrhage, stroke, or traumatic brain injury. Given the sensitivity of copeptin to brief hypoxic episodes in animal models, copeptin might also be of diagnostic value in unclear clinical situations such as apneas or apparent life threatening events.

Together, the availability of quantitative copeptin measurements opens new avenues to study copeptin as an adjunct in the diagnosis of various diseases and to further understand AVP action.

## Author Contributions

SW conceived and designed the review. KE and SW performed the literature search. KE analyzed the data and prepared the graphs. SW drafted the initial manuscript. KE and SW critically revised the manuscript for important intellectual content, agreed on the final manuscript, and approved its submission for publication.

## Conflict of Interest Statement

The authors declare that the research was conducted in the absence of any commercial or financial relationships that could be construed as a potential conflict of interest.
